# investigation of nacre nanostructure by analyzing its structural color pattern

**DOI:** 10.1038/s41598-021-99327-4

**Published:** 2021-10-04

**Authors:** Nicole Fan, Chunhui Zhou, Elina Myagkaya

**Affiliations:** New York Laboratory, Gemological Institute of America (GIA), New York, 10036 USA

**Keywords:** Biophysics, Mathematics and computing, Nanoscience and technology

## Abstract

Produced by light interacting with structures at micrometer or nanometer levels, structural color can be utilized to investigate details of material structures. In this article, we studied the pattern of interference color generated from repetitive aragonite–conchiolin double layers on colorless nacreous pearls. Based on qualitative wave analysis and quantitative electromagnetic computation, we theoretically concluded such patterns are mainly determined by aragonite layer thickness. We also demonstrated how to predict the aragonite layer thickness and estimate the conchiolin refractive index variation on a Tahitian pearl with near-colorless body color and strong iridescence. We believe this approach offers a new perspective to study nanostructures in biology and mineralogy.

## Introduction

**Figure 1:**
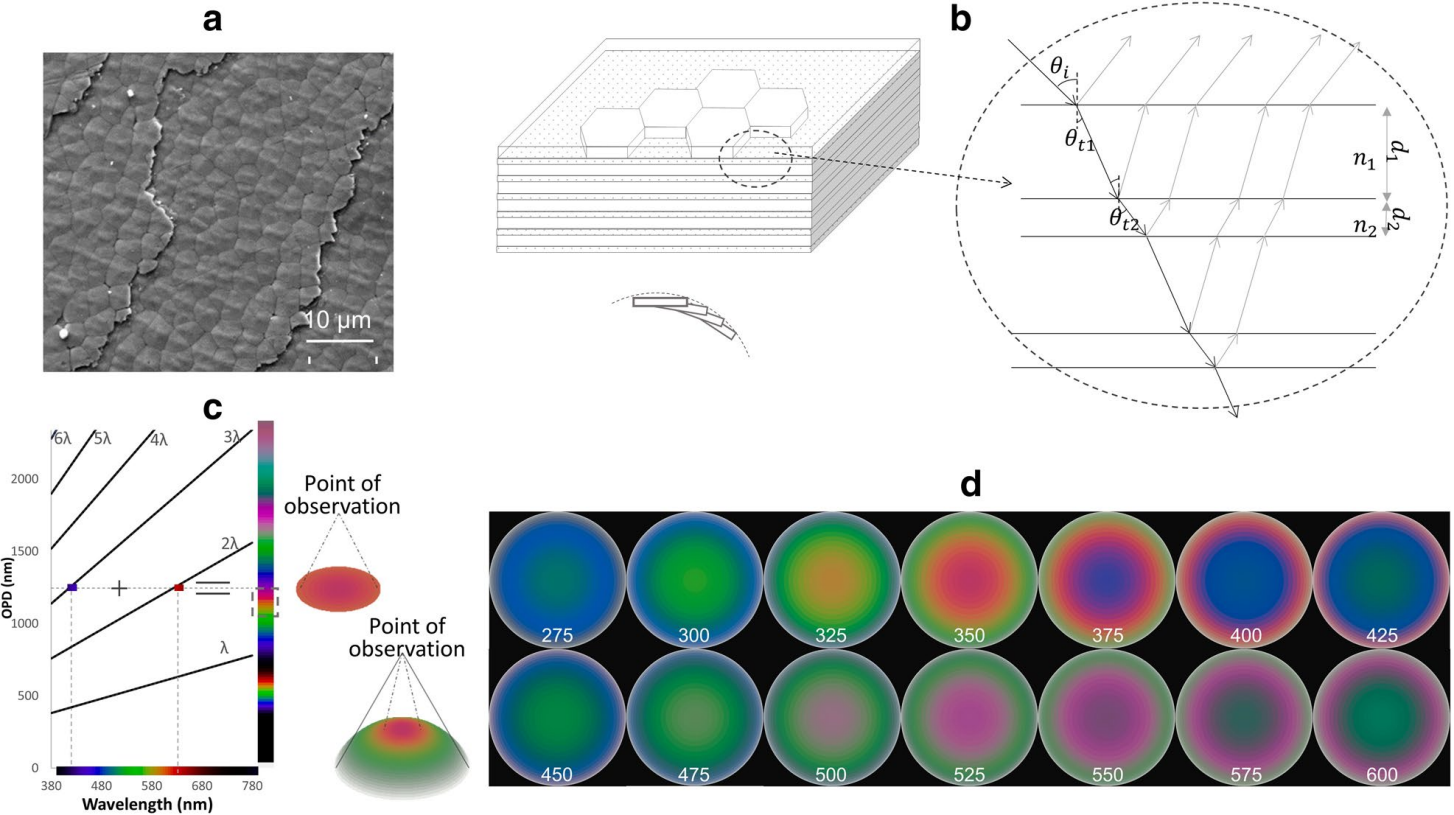
(**a**) An example of nacre’s layered structure with its surface under scanning electron microscope. (**b**) Cross-sectional illustration of aragonite–conchiolin double layers and the staggering of these layers due to surface curvature. (**c**) Quick estimation of repetitive double layers’ interference color and color pattern following Bragg’s Law. (**d**) Interference pattern simulations on a round pearl with aragonite layer thickness varying from 275 to 600 nm.

Structural color is produced by the interaction of visible light with nanostructure, so it is in turn an indication of the structure details^1^. As visual perception of light, color appearance of an object would vary with illumination, yet objects with different spectral properties could possibly match in color^2^. On the other hand, spatial distribution of the structural color retains no matter how illumination changes, and its associated color pattern minimizes the confusion caused by metamerism. Therefore, we focused on structural color pattern analysis to investigate nacre structure in this article. Nacre is a bio-mineralized composite secreted by mollusk in the forms of either shell or pearl. Nacre’s characteristic brick-and-mortar structure is composed of ~ 0.5 μm thick and ~ 10 μm wide aragonite tablets bonded by ~ 25 nm thick organic conchiolin sheets^3–8^ (Fig. 1a). Scanning electron microscopy (SEM) and atomic force microscopy (AFM) have been extensively used to investigate this material. Stacking with crystallographic c-axis perpendicular to nacre surface, aragonite tablets could form submicroscopic aragonite layers. In addition, these flat layers slightly stagger in alignment with the surface curvature at macro scale (Fig. 1b). Thus, nacre normally holds two sets of repetitive structures: the aragonite–conchiolin double layer, from which interference occurs; and the oriented surface grooves made up of aragonite layers’ edges, acting as diffraction gratings^4,9–11^. Although the diffraction fringes on nacre surface indicate the numerical value of groove spacing and the blaze angle, such a pattern is normally too delicate to be distinguishable with angular alteration from surface curvature. On the other hand, the interference equation (Eq. 1) derived by Bragg’s Law shows that the angular position of the interference color is much less variable. The left expression in the equation is optical path difference (OPD) arising from the aragonite–conchiolin double layer, where n is the refractive index (RI), d is the submicroscopic layer thickness, with subscripts 1 and 2 referring to media aragonite and conchiolin, respectively. θ_t_ is the angle of transmission and could be calculated by the angle of incidence θ_i_ using Fresnel Equations. Light waves keep full in phase when their OPD is integer (m) times of the wavelength λ. At normal incidence, θ_t_ = θ_i_ = 0 and n_1_ considered as 1.685 at aragonite’s c-axis^12^, Eq. (1) could be written as Eq. (2). If we set d_1_ = 350 nm, n_2_ = 1.3, and d_2_ = 25 nm based on previous studies of nacre^3,5–13^, then OPD can be calculated as 1244.5 nm. Draw assisting lines at slope m (m = 1, 2, 3…), a horizontal line at 1244.5 nm high intersects with the assisting lines of slopes 2 and 3 at 622.25 nm and 414.83 nm respectively. As 622.25 nm is the dominant wavelength of red, and 414.83 nm, blue, the two light waves additively mix to produce magenta color (Fig. 1c). If θ_i_ increases, the color will change from magenta to orange, yellow, and green with a gradual decrease in OPD. When viewing angle increases, angle of incidence increases much faster on curved surface, so the corresponding interference pattern will be more distinctive. Given d_1_ is the dominant factor in OPD expression, we expected that aragonite layer thickness could be estimated by interference pattern observed on round pearl.

1 $$ 2\left( {n_{1} d_{1} \cos \theta_{t1} + n_{2} d_{2} \cos \theta_{t2} } \right) = m\lambda $$

2 $$ 2\left( {1.685d_{1} + n_{2} d_{2} } \right) = m\lambda $$

Because the interference equations cannot take amplitude variations into consideration, we switched to electromagnetic method to compute the theoretical reflectance^14^, and calculated the color perceived by the CIE standard observer^2^. Such a color perception is described by CIE lightness (L), chroma (C), and hue (H). As tone, saturation, and hue are extensively used to describe the same perceptual properties, we defined tone as lightness, saturation as the ratio of chroma to lightness in this article, to be in line with definitions in CIE color systems. A series of LCH values associated with the viewing angle should sufficiently represent an interference pattern (Table S1 in Supporting Information). To visualize the numerical description, we further synthesized CIELCH colors via 8-bit computer graphics, and generated series of interference pattern simulations on round colorless pearl, with aragonite layer thickness ranging from 275 to 600 nm at 25 nm increment (Fig. 1d).

**Figure 2:**
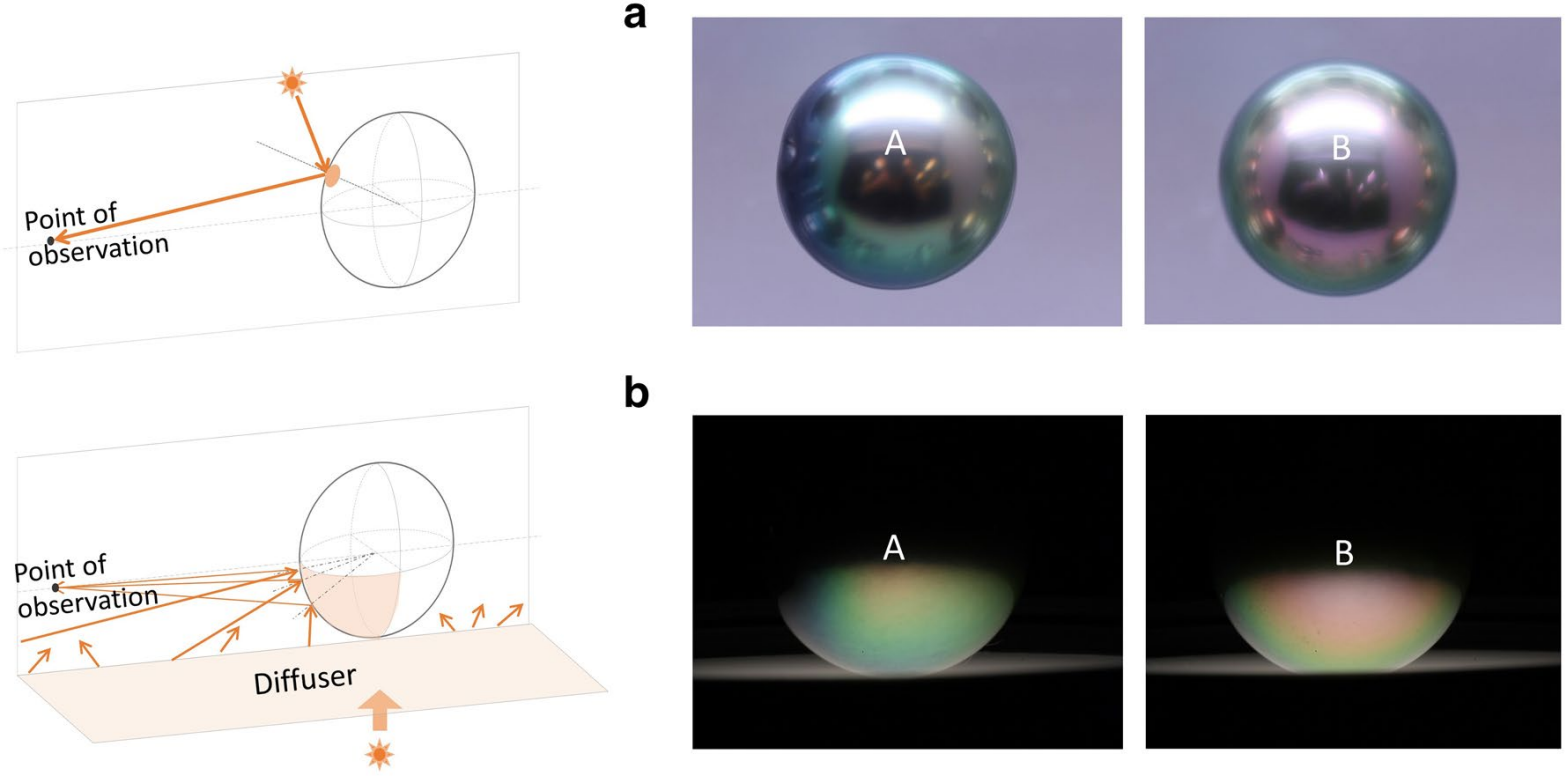
A round Tahitian pearl observed under (**a**) overhead lighting, and (**b**) interference enhanced setup.

Under normal lighting and viewing conditions, a glossy round pearl would show a bright speckle from directional lighting, and appear dark at center due to light being blocked by the observer (Fig. 2a). We used diffused lighting shone from the bottom to observe the concentric pattern on the lower half of the pearl (Fig. 2b). The photographs were taken from two opposite sides (A and B) of one Tahitian pearl. The two concentric patterns in Fig. 2b are close to the simulations where d_1_ = 325 nm and d_1_ = 350 nm in Fig. 1d. In order to validate our theoretical predications, we need to conduct destructive analysis on nacre to see whether the differences in iridescent patterns correlate with differences in aragonite layer thickness.

## Results

**Figure 3:**
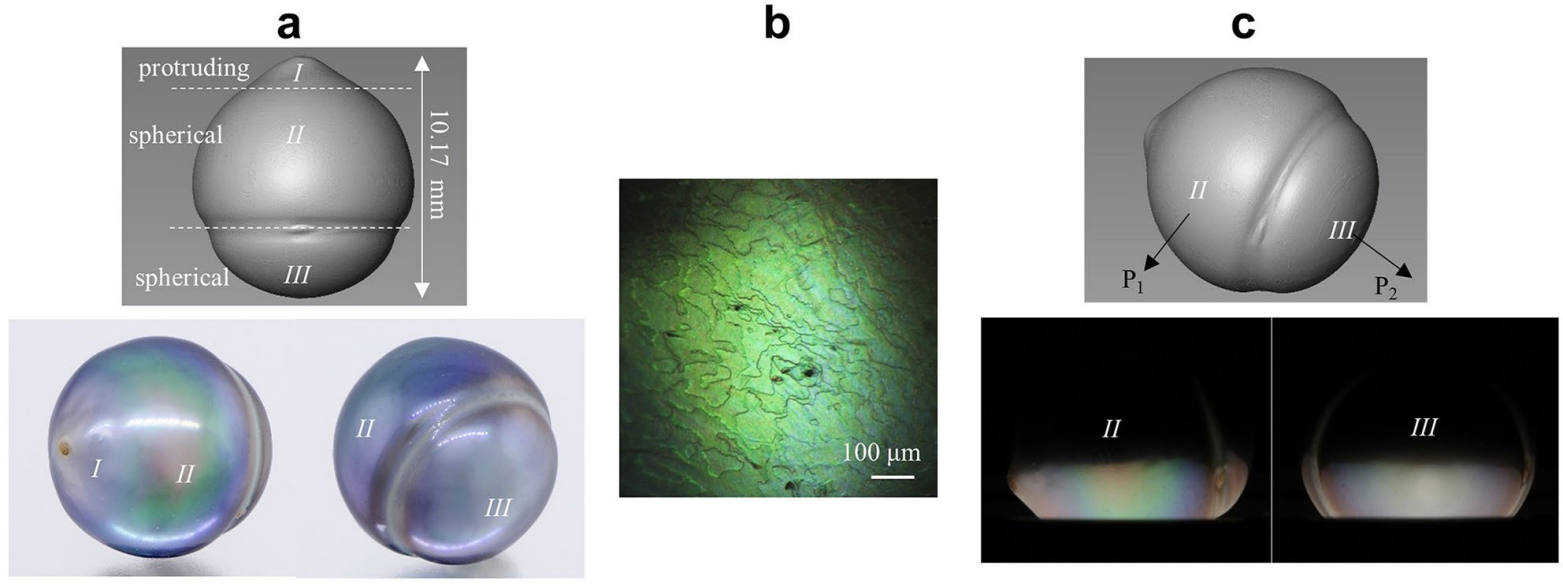
The sample Tahitian pearl used in destructive analysis. (**a**) Its 3D surface model (top) and photographs taken in jewelry lighting box. (**b**) Its surface features observed under optical microscope. (**c**) Its interference patterns observed at points P_1_ and P_2_.

A drop shaped Tahitian pearl with an irregular protruding tip was selected for destructive nacre analysis (Fig. 3a). Its light grey body color ensures the influence from pigment absorption would be appreciably small. Its RI was recorded to be in the range of 1.5–1.68 using a conventional refractor. Its dimensions were 9.31 × 9.39 × 10.17 mm. It was composed of three major parts in terms of shape, from top to bottom: I protruding, II and III spherical, where part II and part III showed strong and slightly different iridescences. Under optical microscope and overhead lighting the pearl’s surface showed yellowish green color, with aragonite layers’ edges appearing as multiple random wavy lines (Fig. 3b). The lack of oriented lines excluded the diffraction effect on this sample surface, so the yellowish green color could only come from thin film interference with a touch of pigmentation. As mentioned in “Introduction” part, individual interference color is not sufficiently conclusive to nacre structure details. We put the sample under interference enhanced setup and observed the color patterns on part II and part III (Fig. 3c). The two concentric patterns suggested the aragonite layers were in 300–325 nm thickness range, and slightly thicker at part II. In addition, a less saturated color pattern on part III implied a relatively higher conchiolin RI according to the wave analysis in Supporting Information.

**Figure 4:**
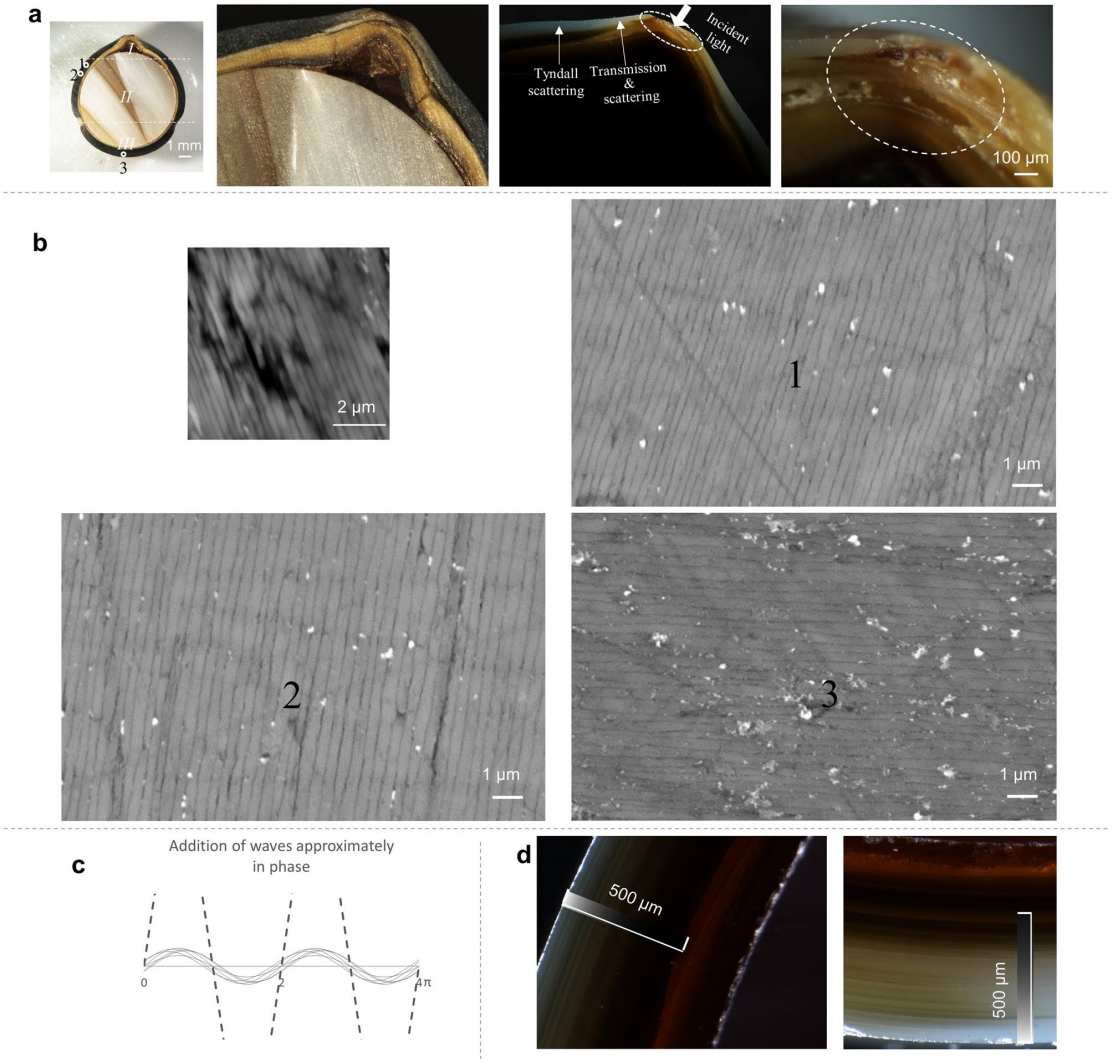
Cross sectional analysis on sample’s nacre. (**a**) Observations under optical microscope from left to right: an overview of cross section of the sample, the protruding tip under reflected and transmitted lighting, and the protruding tip under higher magnification. (**b**) BSE images at 1 nA probe current (top left), and 500 pA probe current on different spots of the sample. (**c**) Superposition of approximately in-phase waves. (**d**) Light impinges in nacre much deeper at area 3 (or part III).

The sample was then sawn into two equal halves. Its cross-section exhibited an approximately 0.5 mm-thick nacre surrounding a round bead nucleus, superimposed by a layer of thin and light toned calcite sheet, under an optical microscope with overhead lighting (Fig. 4a, 1st and 2nd from left). Because the color observed from nacre surface is light toned, the dark toned color observed on nacre’s cross-section should not come from the pigment absorption. Instead, it indicates that light can barely reflect from the lateral surface of the aragonite tablets. So we switched the lighting to the transmitted mode by pointing the fiber optic at the chipped area of the protruding tip from the side, and the nacre beneath the surface appeared translucent with slightly blue tint (Fig. 4a, 3rd from left). The blue tint was assumed to be caused by Tyndall Scattering and the nacre should contain the same type of organic pigments as those disseminated within the light toned calcite^13^. We increased the magnification and tilted the tip slightly, and as a result, the color appearance was consistent in hue (brown) on both nacre and calcite layers, but greatly differed in saturation due to pigment concentration variation (Fig. 4a, right). The nacre beneath the surface was almost colorless, which suggests the simulations with non-pigmentation could apply well on the sample.

One half of the sample was polished and gold coated for SEM analysis on a Zeiss EVO MA 10 tabletop-type scanning electron microscope. Under the backscatter detector, 15 kV accelerating voltage, and 1 nA probe current, we could observe that aragonite layers space at approximately 330 nm (d_1_ + d_2_) (Fig. 4b, top left). With a deduction of conchiolin layer thickness, assumed 25 nm, the aragonite layer thickness is approximately 305 nm, within the range estimated by interference color patterns, 300–325 nm. The SEM image also revealed the aragonite layers stagger more in highly curved pearl’s nacre than they do in shell’s relatively flat nacre, with noticeable thickness variance. We switched to 500 pA probe current so more consecutive layers could be simultaneously present. Multiple spots were scanned and Fig. 4b shows SEM images from spots at areas 1, 2, and 3 marked in Fig. 4a. The lines formed by the layers’ edges spread out at specific densities, with some layers slightly ‘thicker’, and others ‘thinner’ at nanometer level. The corresponding phase difference variance should average out upon superposition (Fig. 4c), so the hue of the interference color should not be affected, while the lightness and chroma might be reduced a little. Overall, the impact to the interference color from the thickness variance is negligible. In addition, the lines at area 2 are slightly less dense than that at area 1 and 3, which is in line with what was implied by the previously observed hue pattern difference. We hypothesized that aragonite tablets gradually grew thicker from area 1 to area 2, given that nacre structure tend to be evenly organized in space during bio-mineralization^13^.

Next, we checked the uncoated half under transmitted lighting again to compare the light impinging difference at part II and part III, following the implication from saturation difference. And indeed light does impinge in much deeper at part III than it does at part II (Fig. 4d), which correlates with a higher conchiolin index at part III.

## Discussion

Our nacre study based on interference color pattern analysis on colorless pearl was confirmed to be effective. From the difference of calculated spectral reflectance shown in Fig. S1, we hypothesized that pigmentation would considerably alter the color around the hemisphere edge, but the pattern near center area would be much less influenced. So we made a few extra simulations corresponding to a collected golden pearl’s absorbance (Fig. S4). We changed relative absorption intensity to simulate the different pigment concentrations according to Beer’s Law^2^. As a matter of fact the absorbance directly collected from nacre surface is not accurate with the distraction from structural coloration. We plan to measure the relative absorption intensity on grounded nacre powder for the follow-up investigation. We expect the interference pattern approach would become applicable to colored nacreous pearls after destructive experiments on more samples are conducted.

We demonstrated how to non-destructively speculate the aragonite layer thickness by interference color pattern. Following the indications from the iridescence differences, we further confirmed the slight variations in aragonite layer thickness and conchiolin refractive index using optical and scanning electron microscopes. We believe such an approach not only provides a convenient alternative to some sophisticated experiment setups, but also could play an important role for further investigating nanostructure details. Take conchiolin layer thickness for example, although we didn’t check its actual value due to the resolving power of our electron microscope, a reasonably assumed value served well in this study. If we plan to measure conchiolin layer thickness, its influence on the interference color saturation could provide some useful hint, according to which we could design the experiment more effectively.

## Methods

### Calculating and simulating interference pattern

**Figure 5:**
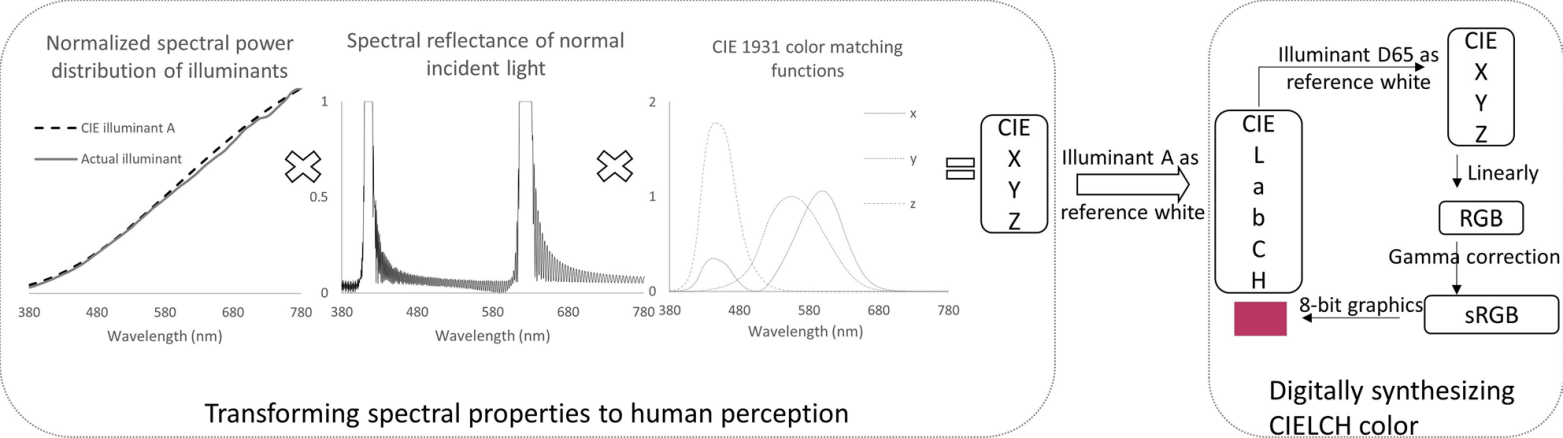
Procedures of color calculation (left) and digital synthetization (right).

For linearly polarized wave impinging on a thin dielectric film case, we can apply boundary conditions on the two adjacent boundaries in both electric and magnetic fields, in order to get the characteristic matrix of the film. Then the amplitude coefficient of reflection and transmission could be calculated accordingly. For any overlaying films, it could be computed by multiplying the characteristic matrix of each film. As the ratio of energy reflected to the incident, reflectance could be derived as the square of coefficient of reflection. According to Malus’ law, the spectral reflectance associated with randomly polarized waves from general light source is the average of orthogonal polarizations p- and s-^2^. Restricted by the coherent length of general light sources, the submicroscopic layers involved in the interference might reach several dozen micrometers deep, referring to Young’s double slit experiment. For the convenience of mathematical treatment, the light was treated completely coherent or completely incoherent, and an arbitrary figure of 200 submicroscopic layers (100 aragonite–conchiolin double layers) was used in the calculation. The spectral reflectance in Fig. 5 was calculated with the same parameters as the ones used in previous qualitative analysis, where d_1_ = 350 nm, n_1_ = 1.685, d_2_ = 25 nm, and n_2_ = 1.3.

To estimate the human perception of received light, we used color matching functions of the 2° 1931 CIE standard observer x, y, z^2^. Considering the abundance of pearls with near colorless (white or light grey) nacre, we treated light as if it simply undergoes reflection and transmission, and would discuss the impact from pigment absorption later. The CIE tristimulus values XYZ of the color could be generated by integrating light source spectrum, spectral reflectance, and color matching functions (Fig. 5). Shown as the dotted line at illuminant spectra, CIE illuminant A was adopted in calculation to match the actual filament lighting. The CIEXYZ were then transformed to CIELab/LCH with illuminant A as reference white (X_w_ = 109.85, Y_w_ = 100, and Z_w_ = 35.58) (Equations S1 and S2). Table S1 lists the CIELCH values of the theoretical interference color with angle of incidence increasing at 5° increment and its first data column corresponds to the calculation illustrated in Fig. 5.

We could digitally synthesize the calculated color by transforming LCH to sRGB, a standard RGB color space for computer graphics and using ITU-R BT. 709 primaries. Because sRGB is based on D65 or D50, D65 was used as reference white (X_w_ = 95.05, Y_w_ = 100, and Z_w_ = 108.9) for a reverse transforming from CIELab to CIEXYZ (Equation S3), which could then be linearly transformed to the values of ITU-R BT. 709 primaries (Equation S4). Next, we applied a non-linear gamma correction to transfer the linear RGB to sRGB (Equation S5). The values of primaries at sRGB space were scaled to 0–255 for 8-bit graphics.

### Interference pattern deviations analysis

**Figure 6:**
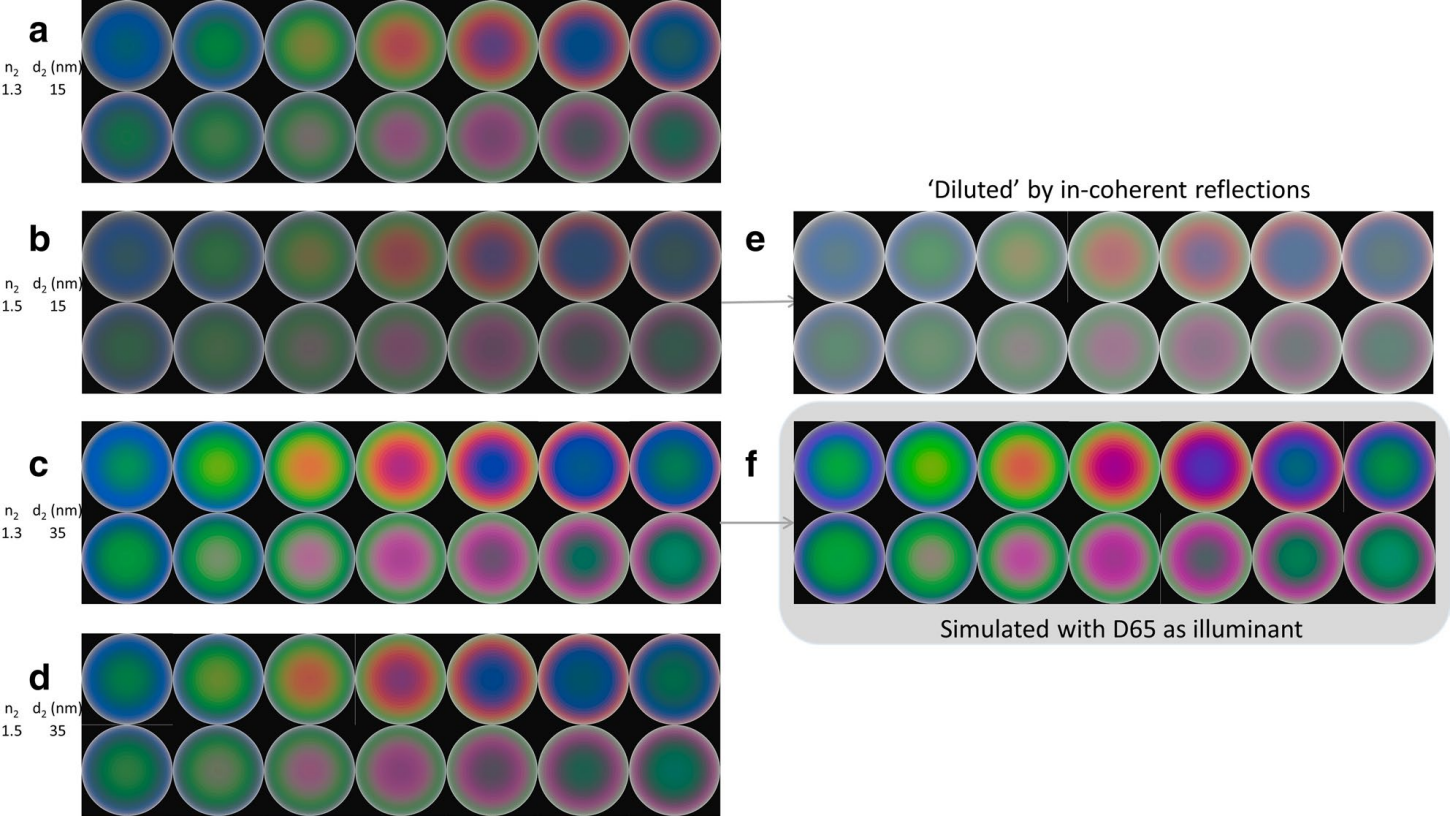
Interference pattern simulations with (**a**)–(**d**) showing the possible hue patterns; (**c**) the high saturation associated with thicker conchiolin layer; (**e**) the low saturation associated with lower conchiolin index; and (**f**) similar patterns with D65 as illuminant.

According to Eqs. (1) and (2), we hypothesized that interference pattern could be used to estimate the aragonite tablet thickness of nacreous pearl. Conchiolin layer is extremely thin so its thickness and index (d_2_, n_2_) can hardly be accurately measured. We generated the simulations with the range of these two parameters showing the possible interference pattern deviations in terms of hue (Fig. 6a–d), with the approximation of n_1_ being constant 1.685.

With an additional insight into the interaction between light waves and the repetitive double layers (Supporting Information Section 2), we concluded that slightly thicker conchiolin layer results higher chroma in color, and higher conchiolin index results lower saturation. So Fig. 6c shows the highest chroma and Fig. 6e illustrates a possibly lowest saturation with an imaging processing of whiteness enhancement as the ‘dilution’ from the wavelength-independent reflection.

As was hypothesized and theoretically confirmed that the interference pattern on round nacre surface is pretty sustainable to slight nanostructure variations. We further showed its sustainability under different illumination condition by simulating the patterns with illuminant D65 in Fig. 6f, which is the only series with illuminant D65 in this article.

### Interference pattern observing and recording

To create the bottom diffused lighting illustrated in Fig. 2b, we stayed in a darkroom and put a glass diffuser on the well-light of a gem microscope, which used filament bulb. The light spectrum was collected above the diffuser with a MK350N Premium spectrometer (solid line in left of Fig. 5). We initially used a 16 mm lens to photograph the interference pattern in front of the pearl at 25 cm distance, to mimic the viewing geometry of human eyes. It turned out the pearl in the image is too small to show the details, so we switched to a 90 mm macro lens given that the deviation from the changing viewing geometry is appreciably small. The white balance was set at Incandescent light mode. The ISO was set at 200. The f-number was set at 32 under aperture priority mode, so the camera’s virtual entrance was at its smallest to reduce the color blending (blurring) from multiple incident angles.

## Supplementary Information

Supplementary Information.
