# Supplementary material for: investigation of nacre nanostructure by analyzing its structural color pattern
Source: Sci Rep. 2021 Oct 4;11:19686. doi: 10.1038/s41598-021-99327-4 (PMC8490444; doi:10.1038/s41598-021-99327-4)
Supplement: Supplementary file 1 — Supplementary Information. [file 41598_2021_99327_MOESM1_ESM.pdf]

Supporting Information

INVESTIGATION OF NACRE NANOSTRUCTURE BY ANALYZING ITS STRUCTURAL COLOR PATTERN

Nicole Fan[1*], Chunhui Zhou[1], and Elina Myagkaya[1]

[1]New York Laboratory, Gemological Institute of America (GIA), New York, 10036, U.S.
*nfan@gia.edu

1. Color spaces transforming formulas and examples

Equations (S1)-(S5) are transforming formulas used in the article to present the CIELCH color by 8-bit computer graphics [1].

$$\begin{aligned} L^* &= 116(Y/Y_W)^{1/3} - 16 \\ a^* &= 500\left[(X/X_W)^{1/3} - (Y/Y_W)^{1/3}\right] \\ b^* &= 200\left[(Y/Y_W)^{1/3} - (Z/Z_W)^{1/3}\right] \end{aligned} \qquad (S1)$$

S1. *Equations for transforming CIEXYZ to CIELab*

$$\begin{aligned} C^*_{ab} &= (a^{*2} + b^{*2})^{1/2} \\ h_{ab} &= tan^{-1}\left(\frac{b^*}{a^*}\right) \end{aligned} \qquad (S2)$$

S2. *Formulas to convert Cartesian coordinates (a*, b*) to polar coordinates (C*, h)*

$$\begin{aligned} X &= X_W\left(\frac{L^*+16}{116} + \frac{a^*}{500}\right)^3 \\ Y &= Y_W\left(\frac{L^*+16}{116}\right)^3 \\ Z &= Z_W\left(\frac{L^*+16}{116} - \frac{b^*}{200}\right)^3 \end{aligned} \qquad (S3)$$

S3. *Equations for transforming CIELab to CIEXYZ*

$$\begin{bmatrix} R \\ G \\ B \end{bmatrix} = \begin{bmatrix} 3.2406 & -1.5372 & -0.4986 \\ -0.9689 & 1.8758 & 0.0415 \\ 0.0557 & -0.2040 & 1.0570 \end{bmatrix} \begin{bmatrix} X \\ Y \\ Z \end{bmatrix} \qquad (S4)$$

S4. *Linear transforming matrix from CIEXYZ to ITU-R BT. 709 primaries*

$$\gamma(u) = \begin{cases} 12.92u & u \le 0.0031308 \\ 1.055u^{1/2.4} - 0.055 & otherwise \end{cases} \qquad (S5)$$

S5. *Gamma correction from linear RGB to sRGB*

Below table lists the data generated with above equations.

θ_i (°)	0	5	10	15	20	25	30	35	40	45	50	55	60	65	70	75	80	85
*L** =	44.8	45.0	45.9	47.5	49.3	51.6	53.7	55.4	56.6	56.8	56.3	55.6	55.1	55.7	58.1	63.5	72.0	84.0
*C** =	55.9	56.0	56.2	56.7	55.8	53.7	49.1	44.1	43.1	46.1	50.0	50.2	44.8	34.8	22.6	13.6	7.4	3.2
h_{ab} =	5.2	6.8	10.3	16.4	23.9	33.8	47.1	66.4	90.5	112	127	137	143	146	148	148	142	137

R	187	188	192	197	200	201	194	179	157	130	103	83	78	89	111	137	168	206
G	55	56	59	64	73	87	103	119	134	142	147	148	146	145	148	159	179	210
B	99	97	94	88	81	74	67	62	58	57	61	70	83	98	118	140	168	205

Table S1. *CIELCH values corresponding to the reflectance with increasing incident angle; and RGB values for 8-bit computer graphics.*

In Fig. S1a, we plotted the electromagnetically computed reflectance, which was used to calculate the CIELCH colors in above table. Fig. S1b shows the position of the series colors at cylindrical CIELCH space. With a schematic demonstration on how to fill in the cylindrical space with colors of 8-bit computer graphics (Fig. S1c), we synthesized the series colors in Fig. S1d. As no real color gamut could fit in the numerical CIE color space, Fig. S1c doesn't reflect the real mapping from sRGB to CIELCH. With some inevitable distortions, it is still sufficient for illustrative purpose.

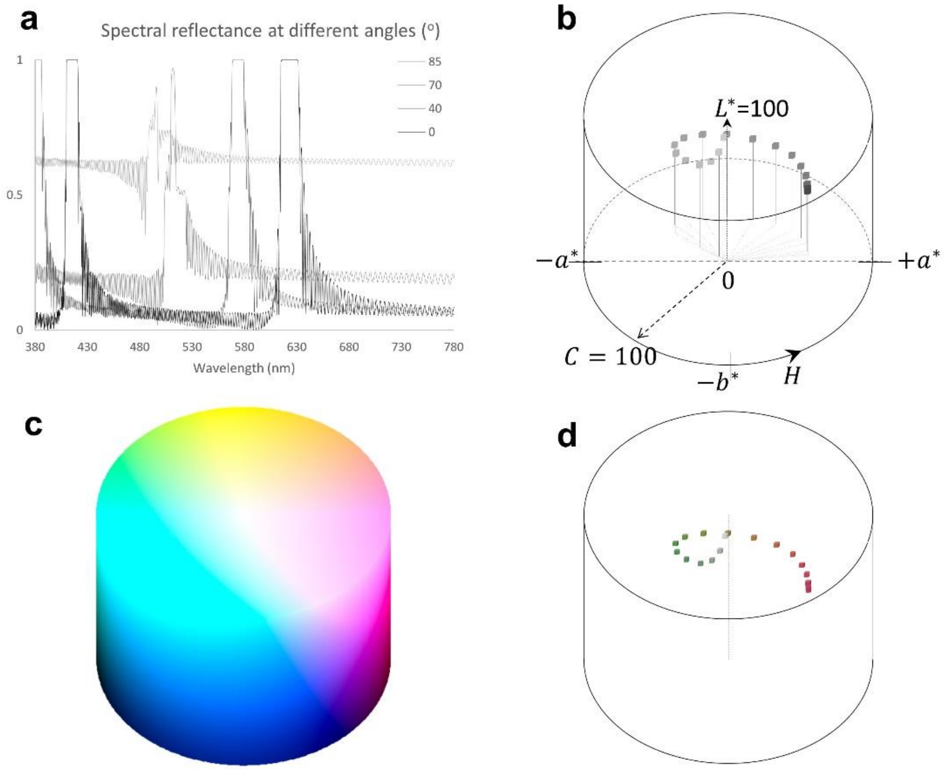

Figure S1. *(**a**) Electromagnetically computed reflectance corresponding to the calculated colors in the table. (**b**) (**c**) (**d**) Synthetization of the series CIELCH colors.*

2. Insight into light waves propagating and interacting with nacre's multiple double layers

Assume a monochromatic wave $\tilde{E}_i$ incident on two media's boundary, part of it reflects back as $\tilde{E}_r$, while the remaining portion transmits as $\tilde{E}_t$ (Fig. S2a). $\tilde{E}_r$ and $\tilde{E}_t$ could be expressed as $r\tilde{E}_i$ and $t\tilde{E}_i$, where r and t are amplitude coefficient upon reflection and transmission. r and t could be calculated via Fresnel Equations at p- and s- polarizations (parenthesized in equations S6) [2]. While t is always positive, r could be either positive or negative, with minus sign indicating a π phase shift. The absolute value of r is relatively very low, so we could focus on the light scattering backwards upon 1st reflection at each interface for multiple boundaries ($\tilde{E}_{1r}$, $\tilde{E}_{2r}$, …)(Fig.

S2b). The RI and layer thickness are alternative (n_1, d_1) and (n_2, d_2) for nacre structure. We set n_0=n_2 for the simplicity of mathematical treatment, so the amplitude coefficient could be notated as (r_{12}, t_{12}), and (r_{21}, t_{21}) on every other interface. As r_{12}=-r_{21} due to a π phase shift between the internal and external reflections, the reflected waves could be expressed by Equations (S6), where **Λ** is the OPD associated with each submicroscopic layer, and λ_0 is the wavelength in vacuum. The waves could be grouped by one reflected from conchiolin/aragonite interface, and the other, aragonite/conchiolin interface. The waves at each group sustain the same phase difference associated with the sum OPD of aragonite-conchiolin double layer. As is illustrated in Fig. S1c, the waves would enhance when their phase difference is integer multiple of 2π, otherwise, they average out.

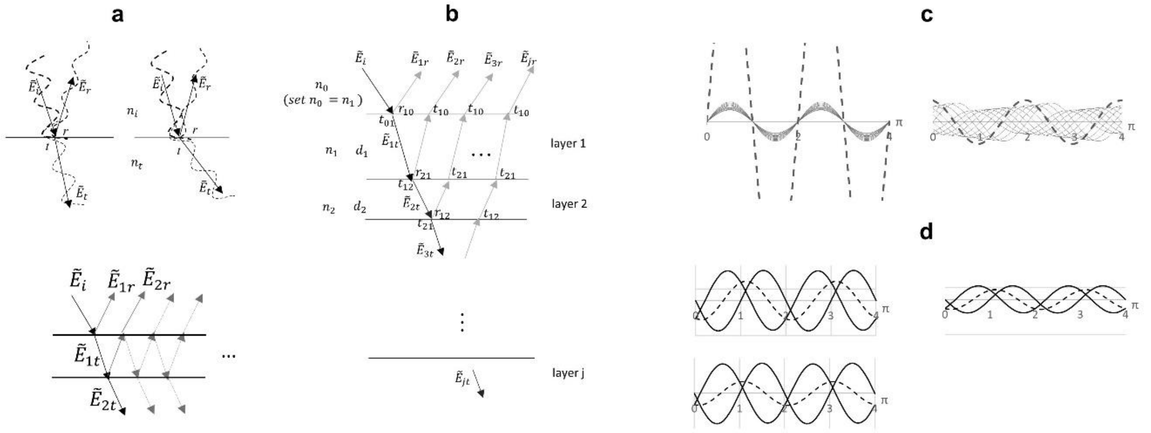

Figure S2. *Schematic illustration of (**a**) light incident at two media's boundary in the form of p-polarized wave, and (**b**) light incident at consecutive double – layers. (**c**) Superposition of in-phase & out-of-phase waves. (**d**) Superposition of two near out-of-phase waves with slightly varying phase difference and amplitude.*

$$\begin{cases} \tilde{E}_{1r} = r_{12}\tilde{E}_i \\ \tilde{E}_{2r} = t_{21}r_{21}t_{12}\tilde{E}_i = -t_{12}t_{21}e^{i\frac{2\pi}{\lambda_0}\Lambda_1}\tilde{E}_{1r} \\ \tilde{E}_{jr} = (t_{12}t_{21})^2 e^{i\frac{2\pi}{\lambda_0}(\Lambda_1+\Lambda_2)}\tilde{E}_{(j-2)r}, \quad ...j > 2 \end{cases} \quad \begin{pmatrix} r_s = \dfrac{n_i \cos\theta_i - n_t \cos\theta_t}{n_i \cos\theta_i + n_t \cos\theta_t} & r_p = \dfrac{n_t \cos\theta_i - n_i \cos\theta_t}{n_t \cos\theta_i + n_i \cos\theta_t} \\ t_s = \dfrac{2n_i \cos\theta_i}{n_i \cos\theta_i + n_t \cos\theta_t} & t_p = \dfrac{2n_i \cos\theta_i}{n_t \cos\theta_i + n_i \cos\theta_t} \end{pmatrix} \quad (S6)$$

Above is a detailed illustration of how interference equations (1) and (2) were derived following Bragg's Law, which simply reveals the relationship between the nanostructure and the hue of interference color. To further analyze how lightness and chroma vary, we focused on the waves reflecting from one layer's top and bottom interfaces, $\tilde{E}_{(j-1)r}$ and $\tilde{E}_{jr}$. Under Bragg condition, the two waves have a phase difference of either $\pi - \frac{2\pi}{\lambda_0}\Lambda_2$ or $\pi + \frac{2\pi}{\lambda_0}\Lambda_2$; and the superposition of such consecutive two waves keep in-phase, so the irradiance of enhanced light waves highly correlates with the amplitude of such a superposition. As $\tilde{E}_{(j-1)r}$ and $\tilde{E}_{jr}$ are near out-of-phase with a small deviation of $\frac{2\pi}{\lambda_0}\Lambda_2$, a decrease in d_2 results a decrease in amplitude of the superposition (Figs. S2d, left). On the other hand, an increase in n_2 also reduces amplitude of the superposition, as the result of decrease in individual amplitude (Figs. S2d, right). In summary, either a decrease in d_2 or an increase in n_2 would reduce the lightness and chroma of interference color, and vice versa. In addition, such a qualitative analysis could be quantified by the electromagnetic method, with reflections of all orders taken into account.

The discussion so far hasn't taken coherent length into consideration. The coherent length of the light source we generally encounter is unmeasurable, but we can qualitatively estimate when reflecting light becomes incoherent based on an approximate range. This range is several dozens of micrometers according to Young's experiment. For the wave propagating forward, $\tilde{E}_t$ suffers amplitude decay every time it advances through two media's boundary, as indicated by the transmittance coefficient t. And the decay doubles on the reflecting wave, as expressed at

equations (S7). In terms of irradiance, I is square of the amplitude (equations S8). The irradiance of the light reflecting from layer j keeps exponential decay with $(t_{12}t_{21})^2$ as the base, which equals $[4(n_1n_2)/(n_1+n_2)^2]^2$ at normal incident. Given n_1 is constant and $n_1>n_2$, a greater conchiolin index means slower energy decay of light when it impinges in the repetitive double layers, so higher proportion of incident light could reflect back from deeper layers. The longer the light waves travel, the more likely they become out of coherent length. As wavelength-independent reflection mainly adds lightness to the color, a greater conchiolin index possibly results a less saturated color. We plotted the exponential decay associated with I_{jr} as reference (Fig. S3), with it in mind that I_{jr} is slightly lower than the actual irradiance reflecting at each boundary, which includes the energy from higher orders of reflections.

$$\begin{cases} \tilde{E}_{jt} = (t_{12}t_{21})^{j/2} e^{i\delta} \tilde{E}_i \\ \tilde{E}_{jr} = (t_{12}t_{21})^{j} r_{12} e^{i\delta} \tilde{E}_i \end{cases} \quad j = 2,4,6\,, \ldots \qquad (S7)$$

$$\begin{cases} I_{jt} = (t_{12}t_{21})^{j} I_i \\ I_{jr} = (t_{12}t_{21})^{2j} r_{12}^2 I_i \end{cases} \quad j = 2,4,6, \ldots \qquad (S8)$$

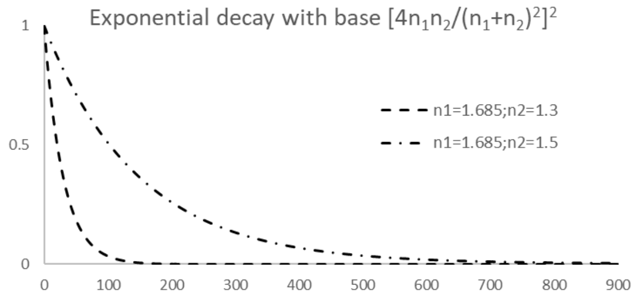

Figure S3. *Exponential decay with different parameters.*

3. Impact from pigment absorption

Because it is not easy to accurately obtain the absorbance, we set various intensities on a spectrum collected from a golden pearl surface to mimic the different pigment concentrations [1]. Fig. 4S shows the manipulated spectra and the corresponding pattern simulations for 350 nm aragonite thickness case.

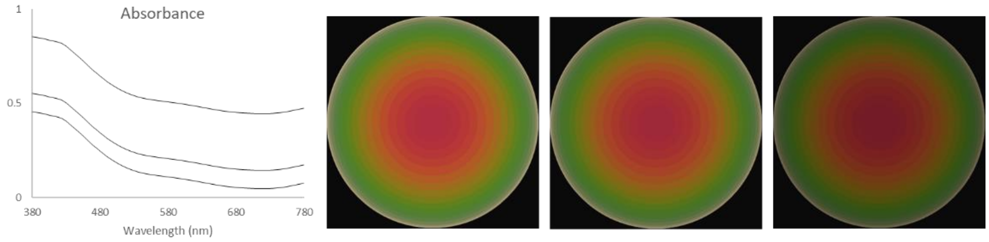

Figure S4. *Interference pattern simulations with assumed absorbance of a golden pearl.*

References

1. Berns, R. S. *Billmeyer and Saltzman's Principles of Color Technology* (3rd ed.) (Wiley-Interscience, 2000).

2. Hecht, E. & Guardino, K. *Optics* (3rd ed.) (Addison Wesley Publishing Company, 1997).
